# Supplementary material for: PHF2-mediated H3K9me balance orchestrates heterochromatin stability and neural progenitor proliferation
Source: EMBO Rep. 2024 Jun 18;25(8):18. doi: 10.1038/s44319-024-00178-7 (PMC11315909; doi:10.1038/s44319-024-00178-7)
Supplement: Supplementary file 1 — Appendix [file 44319_2024_178_MOESM1_ESM.pdf]

Appendix for

PHF2-mediated H3K9me balance orchestrates heterochromatin stability and progenitor proliferation in early neurogenesis

Samuel Aguirre *, Stella Pappa *, Núria Serna-Pujol, Natalia Padilla, Simona Iacobucci, Silvina A Nacht, Guillermo P Vicent, Albert Jordan, Xavier de la Cruz, and Marian A Martínez-Balbás

Table of contents:

Appendix Figure S1________________________Page 2
Appendix Figure S2________________________Page 3
Appendix Figure S3________________________Page 4
Appendix Figure S4________________________Page 5
Appendix Figure S5________________________Page 6
Appendix Table S1 ________________________Page 7
Appendix Table S2 ________________________Page 8
Appendix Table S3________________________ Page 8

APPENDIX FIGURES

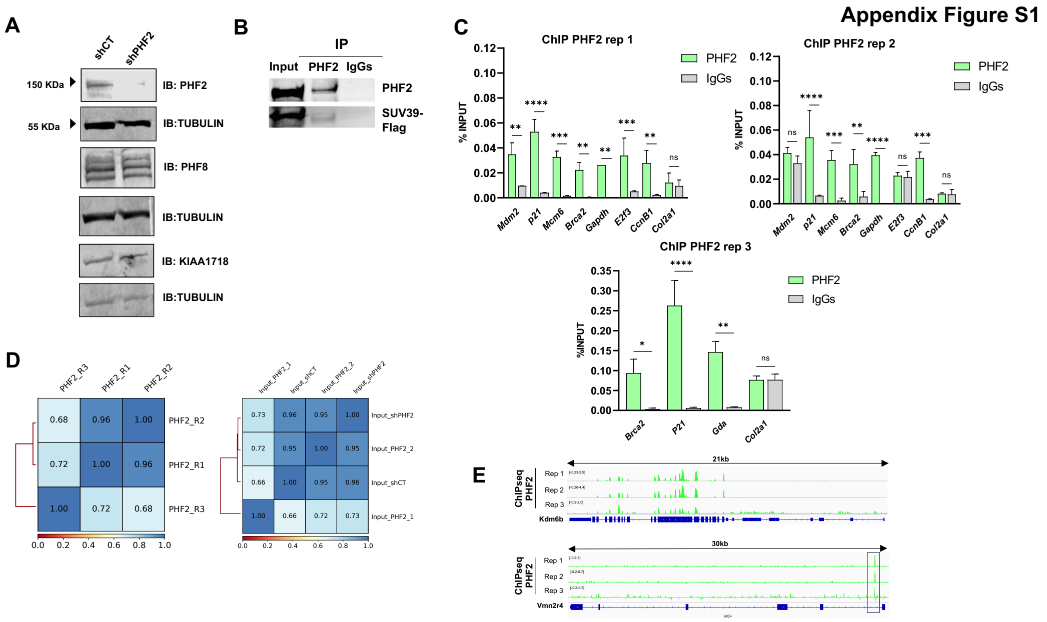

Appendix Figure S1. PHF2 is enriched in satellites repeats

(A) NSCs were infected with lentivirus expressing shRNA control (shCT) or shRNA specific for PHF2 (shPHF2). 48 h after infection, total protein extracts were prepared and the PHF2, PHF8, KIAA1718 and TUBULIN levels were determined by immunoblot. PHF2 immunoblot details is showed in Figure 1, that corresponds to the exact same experiment.

(B) HEK293T cells were transfected using PHF2 and SUV39H1-Flag expressing vector. PHF2 was immunoprecipitated using PHF2 antibody and the presence of SUV39H1 in the immunopellet was determined by immunoblot using FLAG antibody. IgGs were used as negative control. The images are representatives of two independent experiments with similar results.

(C) PHF2 ChIPs in NSCs were analyzed by qPCR at the indicated gene promoters. The *Col2a1* promoter devoid of PHF2 was used as negative control. Data from qPCR were normalized to the input and expressed as % input. Results from three biological

independent ChIP experiments (rep1-3) and technical triplicates are shown. Errors bars represent SD. *p>0.05; **p<0.01; ***p<0.001; ****p<0.0001 (Student's t-test).

(D) Clustered heatmap depicting Pearson correlation of input and PHF2 ChIP-seq datasets. The correlation analysis is conducted based on read coverage within consecutive 10-kb genomic regions.

(E) IGV genome browser screenshots illustrating the continuous input-subtracted quantification of three replicates of PHF2 ChIP-Seq samples within the indicated regions.

Appendix Figure S2

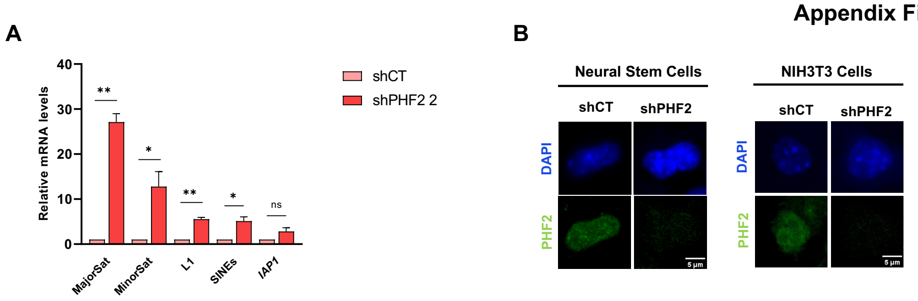

Appendix Figure S2. PHF2 depletion leads to centromeric satellite transcription

(A) RNA levels of major satellite, minor satellite, L1-Lines, Sines and *IAP1* in control (shCT) and PHF2-depleted (shPHF2_2) NSCs were analyzed by qPCR. Data were normalized to *Gapdh* reference gene levels and figure shows values relative to shCT cells. Error bars indicate SD. *p<0.05; **p<0.01 (Student's t-test).

(B) Immunostaining assays of shCT and shPHF2 NSCs (left) or NIH3T3 cells (right). Cells were fixed and stained using anti PHF2 antibody and DAPI to visualize the DNA. They were used as a control of the PHF2 KD. Scale bar indicates 5µm.

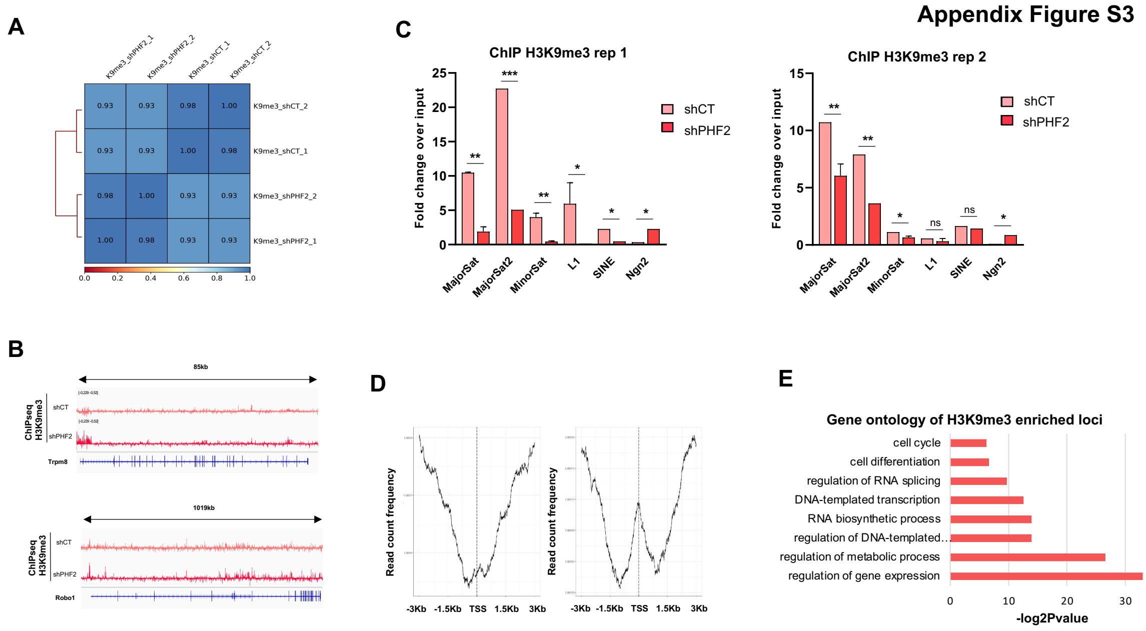

Appendix Figure S3. PHF2 balances H3K9me3

(A) Clustered heatmap depicting Pearson correlation of H3K9me3, ChIP-seq datasets in both shCT and shPHF2 conditions. The correlation analysis is conducted based on read coverage within consecutive 10-kb genomic regions.

(B) IGV genome browser screenshots illustrating the continuous input-subtracted quantification of two merged replicates of H3K9me3 ChIP-Seq samples in shCT and shPHF2 conditions within the indicated genomic regions.

(C) The two replicates (rep1 and rep 2) of H3K9me3 ChIPs in shCT and shPHF2 NSCs were analyzed by qPCR at the indicated genomic regions. Data from qPCR were normalized to the input and expressed as fold change over input; IgG values have been subtracted. Errors bars represent SD. *p>0.05; **p<0.01; ***p<0.001 (Student's t-test).

(D) H3K9me3 read count frequency within genomic bins in control vs PHF2-depleted NSCs around the TSS (+/-3Kb) determined by ChIP-seq.

(E) Gene ontology (GO) analysis showing Biological Process of the H3K9me3 enriched loci upon PHF2 depletion in NSCs, -log2 of p-Value is represented.

Appendix Figure S4

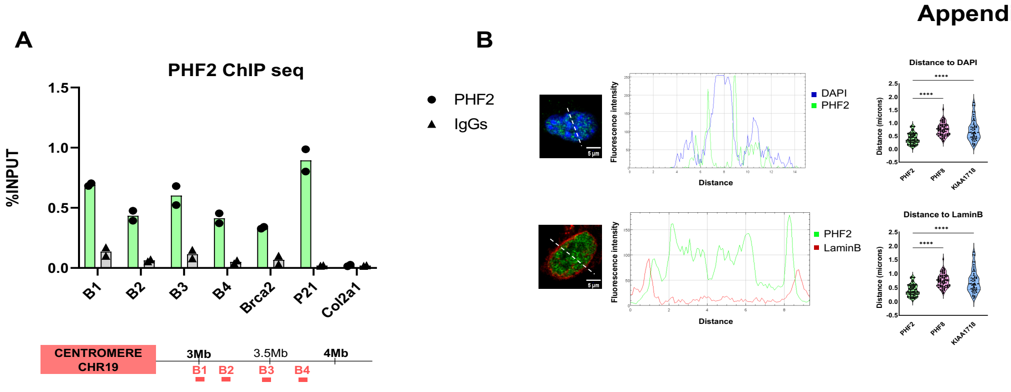

Appendix Figure S4. PHF2 binds PcH boundaries

(A) PHF2 ChIPs in NSCs were analyzed by qPCR within the genomic region spanning positions B1-B4, situated between 3-4 Mb from the centromere of chromosome 19 (see bottom panel). Data from qPCR were normalized to the input. Scatter plot represents the mean of two biological independent experiments. Errors bars represent SD.

(B) Immunostaining assays of NSCs using anti PHF2 and LAMIN B antibodies and DAPI. The distance between PHF2 signal, DAPI and LAMIN B was determined using ImageJ software (see methods). Violin plots represent the distance between PHF2, PHF8 and KIAA1718 signals to DAPI or LAMIN B. Data shown are representative of 3 biologically independent experiments. (n=100). Scale bar indicates 5µm. ****$p<0.0001$ (Student's t-test).

Appendix Figure S5

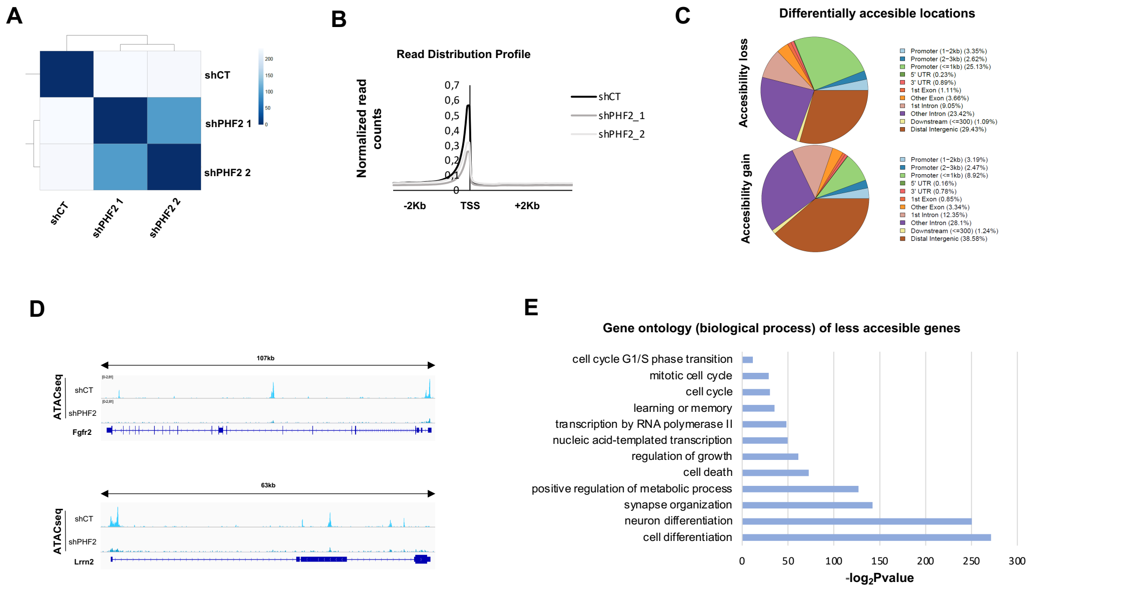

Appendix Figure S5. PHF2 regulates chromatin accessibility

(A) Clustered heatmap depicting Pearson correlation of the two samples PHF2 KD (shPHF2) and control (shCT) ATAC-seq samples based on read coverage within genomic regions.

(B) Read distribution profile of ATAC signal around the TSS (+/- 2Kb) in shCT and shPHF2 NSCs.

(C) Genomic annotation of differentially accessible locations (separated in gain and loss of accessibility) upon PHF2 depletion in NSCs.

(D) IGV genome browser screenshots illustrating the continuous quantification of ATAC-Seq samples in shCT and shPHF2 within the indicated genomic regions.

(E) Gene ontology (GO) analysis showing Biological Process of the of regions that loss chromatin accessibility upon PHF2 depletion in NSCs.

APPENDIX TABLES

Appendix Table S1: PHF2 interactors in NSCs identified by mass spectrometry

Description	ΣCoverage	Score S1
MYH10	7,95	520,82
YBOX1	24,84	231,12
H12	33,49	211,79
PTMA	34,23	184,84
H15	32,29	184,53
PHF2	7,57	180,68
MYH14	2,45	171,89
H14	28,31	166,87
ACTBL	11,97	153,21
YBOX3	9,97	119,67
ROA3	5,80	117,54
LMNB1	3,40	117,45
RL29	21,25	114,43
CASB	8,48	113,01
RL11	14,61	111,40
TBA1C	5,57	102,25
ML12B	11,63	95,35
RS18	12,50	93,79
ATPA	4,52	93,03
RS3A	11,74	88,33
NEST	0,86	87,73
SFPQ	2,15	86,77
RS6	9,64	85,88
1433Z	12,24	85,74
RL8	12,06	84,58
GRN	2,72	80,72
DDX17	4,00	78,12
PAIRB	9,09	77,88
RL34	17,95	72,81
SERA	2,81	72,80
RL36A	16,04	72,58
FHL3	9,00	70,73
RL14	10,14	70,67
FMR1	2,44	67,70
CDV3	10,68	65,88
RL7A	14,29	65,50
GRP78	4,89	64,65
RL19	8,16	64,35
TERA	2,11	62,01
RS2	4,78	61,96
MIC19	3,96	60,44
RS8	6,25	60,26
PDIA3	2,18	57,89
RS19	20,00	57,55
RL23A	17,95	57,46
HMGN1	12,50	57,26
FILA	6,53	56,67
RS14	7,28	55,78
HNRPM	3,29	53,95
RL21	11,25	51,93
HS90B	4,14	51,90
RL4	5,97	51,67

Description	ΣCoverage	Score S1
RS11	12,03	51,47
H10	11,34	50,51
RS26	7,83	49,13
1433E	7,45	48,60
TPM3	7,37	46,36
FABP7	9,09	44,86
TYB10	31,82	43,37
PPIA	10,98	43,13
RRBP1	1,43	42,14
4EBP2	11,67	41,82
RL3	3,47	41,02
RL7	7,41	39,62
RL35	8,94	38,96
RL31	7,20	38,83
SSRP1	1,98	38,38
RS4X	2,66	36,73
RL6	3,04	36,36
EIF3B	2,86	35,70
H4	9,71	35,26
RL24	7,01	34,73
HNRPQ	1,93	34,63
IF2A	3,81	34,46
GKAP1	3,83	34,16
RS3	9,47	33,90
1433F	6,50	33,30
ARPC5	5,96	32,65
RL15	4,41	32,55
NUCB2	1,67	31,35
FIP1	4,13	30,97
CAPR1	1,13	30,94
HP1B3	2,35	30,63
RS30	16,95	30,42
H3C	6,62	29,97
sep-07	2,06	29,89
RS24	6,02	29,51
CNBP	5,62	28,95
RL12	5,45	28,92
NUCL	1,56	28,02
G3P	4,50	26,81
RL28	8,76	26,79
MRCKB	0,35	26,68
BT3L4	3,80	26,60
SERF2	18,64	26,30
RS15	4,83	25,85
RL27	4,41	25,68
SMC2	0,59	25,29
TMOD3	3,69	24,54
FKB1A	12,04	24,32
TEN3	0,77	24,20
CODA1	1,60	23,43
CO1A2	0,66	22,70
RL37A	9,78	22,63

Appendix Table S2: Read count illustrating ChIP-seq mapping statistics

SAMPLE	Read count				
	Raw	Trimmed	NoMap	UniMap	MultiMap
Input_PHF2_NSC_1	39530778	38910461	631676	27001263	11277522
ChIP_PHF2_NSC_1	40037300	40008589	3651163	25142757	11214669
ChIP_PHF2_NSC_2	44950251	44885666	7686267	25330635	11868764
Input_PHF2_NSC_2	25230661	25226125	544955	17902366	6778804
ChIP_PHF2_NSC_3	25237263	25233067	5672044	14327546	5233477
Input_shCT	37213132	37195901	600867	26602478	9992556
ChIP_H3K9me3_shCT_1	38326941	38303957	1060062	22170589	15073306
ChIP_H3K9me3_shCT_2	37214264	37193439	962922	22102556	14127961
ChIP_H3K4me3_shCT_1	26117664	26095069	561088	22607755	2926226
ChIP_H3K4me3_shCT_2	21713993	21706468	434299	18590539	2681630
Input_shPHF2	38223148	38211061	857036	27064714	10289311
ChIP_H3K9me3_shPHF2_1	27508592	27491788	983067	15095082	11413639
ChIP_H3K9me3_shPHF2_2	31462567	31441558	1077711	16941639	13422208
ChIP_H3K4me3_shPHF2_1	35231125	35217137	868704	30126604	4221829
ChIP_H3K4me3_shPHF2_2	38613162	38570893	871551	33145696	4553646
Input_PHF2_NIH	32381050	32348654	626121	21525729	10196804
ChIP_PHF2_NIH_1	33167437	33080914	2044838	20878422	10157654
ChIP_PHF2_NIH_2	33628978	33571057	2059699	21120287	10391071

Appendix Table S3: List of primers used in this study

Gene	Sense Primer	Antisense Primer
Phf2	CCCTGGAGTCTTTCTCACAC	CCGTTCCGATGGATCTTCAAG
MajorSat	TGGCGAGAAAACTGAAAATCACG	TCTTGCCATATTCCACGTCCTAC
MinorSat	AATGATAAAAACCACACTGTAGAACAT	ATGTTTCTCATTGTAACTCATTGATATAC
L1	TGGCTTGTGCTGTAAGATCG	TCTGTTGGTGGTCTTTTTGTC
SINE	GAGCACACCCATGCACATAC	AAAGGCATGCACCTCTACCACC
IAP1	CGCTCCGGTAGAATACTTAC	TGCCATGCCGGCGAGCCTGT
Gda	ACGACGGCACCAAAGAATAC	TTGCCAGACAAGCAATCAAC
Gapdh	ATGTTCGTCATGGGTGTG	CCTTCCACGATACCAAAGTTG
MajorSat (ChIP)	TGGAATATGGCGAGAAAACTG	AGGTCCTTCAGTGGGCATTT
MinorSat (ChIP)	AATGATAAAAACAGTGATTTCG	ATGTTTCTCATTGTAACTCATTGATATAC
Col2a1 (ChIP)	GGTCTCACCGCTCCCTCAT	GCGACCGGGAGCATATAACT
Brca2 (ChIP)	CGGAAACAGACACACACAC	GCAGCGGTAGCTGACTGAC
Mcm6 (ChIP)	ACAGCTTCTGGCATTTCTCG	CCCTGTTATTGGCTGAGGTG
P21 (ChIP)	TAAGGACGTCCCACTTTGCC	GACCTCCTGTGCCTTTACCC
Mdm2 (ChIP)	CCCTGACGCAGGCTTTAGAA	CTGGCAATATTCGTGCTGGC
Gapdh (ChIP)	CGGGATTGTCTGCCCTA	GGAGGTTTCTGCACGG
E2f3 (ChIP)	CCTGGCAGAACACCTGGATT	GCTCTCAGAGGCCAGAAAGG
CcnB1 (ChIP)	TCGGAGGCCTTAAGTCGGTA	GAATCCCAGCTCTTGGCACT
Ngn2 (ChIP	CACAACCTAAACGCCGC	TCTTCGTGAGCTTGGCAT
Cd47 (ChIP)	GGGATGGGAGACTGGTTTGG	CTGACCAGTGGAACCCCATC
Elfn1 (ChIP)	TAGAGAAAGAGCGAGGGTGCT	CTACGCCTACTCAAGCCTCG
Kazn (ChIP)	TCCTTGGGGAGTGGAACTCA	AACAACGGAAGTGCACTGGA
Fgf12 (ChIP)	CCTCTGACCAGGGCTGTGTA	TTGGTGCAGAAATGGGCACTAT
Stat1 (ChIP)	TACACCATACCACGTGCCTG	GCGTGTCCAACCATACCCAG
B1 (ChIP)	GTCACCGCATAGAATGAATTTGG	TAGACAAACTCTCAGCCAAACCAACTGT
B2 (ChIP)	TCCGGATCCACTCTACTCCAATCCTACCT	ACTAAGGGCACATTACTTGTTATGTTTC
B3 (ChIP)	GTTTTGGACATGGCTTTAATGAGTTGGT	TTAAATGCACGGAACGAGAGTCGGC
B4 (ChIP)	ATGGCCAAGAAGGCATCAGTGAG	AATGGCAAGGAGGCGGTGGAATCTG
