# Supplementary material for: PHF2-mediated H3K9me balance orchestrates heterochromatin stability and neural progenitor proliferation
Source: EMBO Rep. 2024 Jun 18;25(8):18. doi: 10.1038/s44319-024-00178-7 (PMC11315909; doi:10.1038/s44319-024-00178-7)
Supplement: Supplementary file 3 — Source data Fig. 1 [file 44319_2024_178_MOESM3_ESM.zip › Figure 1/1A/Blot Anti Tubulin.pdf]

Image ID: 0001782_01
Acquire Time: 22-dic-2022 16:28:22

Image Display Values

Channel	Color	Minimum	Maximum	K
800	Gray Scale (Black on White)	179	2160	0,69

Tubulin

55kDa

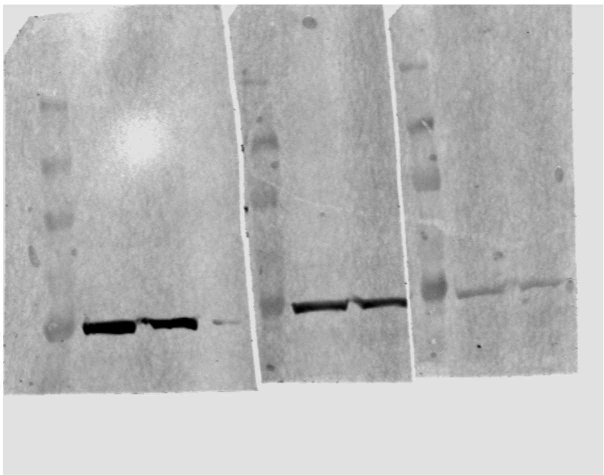

Acquisition Information

Column	Value
Image ID	0001782_01
Acquire Time	22-dic-2022 16:28:22
Channels	800
Resolution	169um
Intensities	7.0
Quality	lowest
Analysis	Manual
Image Name	0001782_01
Comment	
Image Modifications	
