# Supplementary material for: PHF2-mediated H3K9me balance orchestrates heterochromatin stability and neural progenitor proliferation
Source: EMBO Rep. 2024 Jun 18;25(8):18. doi: 10.1038/s44319-024-00178-7 (PMC11315909; doi:10.1038/s44319-024-00178-7)
Supplement: Supplementary file 3 — Source data Fig. 1 [file 44319_2024_178_MOESM3_ESM.zip › Figure 1/1B/Blot 1B.pdf]

LI-COR

Image ID: 0002500_01
Acquire Time: 29-jun-2023 15:22:51

Image Display Values

Channel	Color	Minimum	Maximum	K
700	Gray Scale (Black on White)	59,8	13900	0

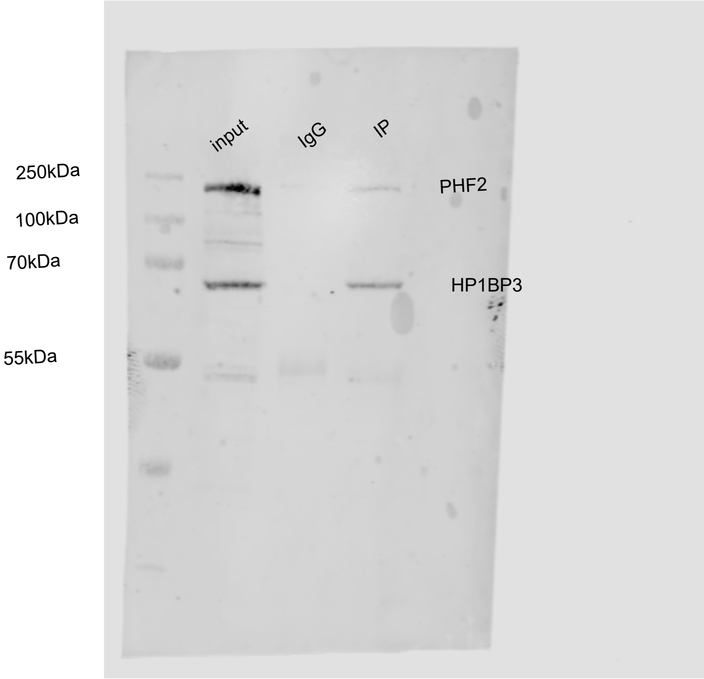

Acquisition Information

Column	Value
Image ID	0002500_01
Acquire Time	29-jun-2023 15:22:51
Channels	700
Resolution	169um
Intensities	5.0
Quality	lowest
Analysis	Manual
Image Name	0002500_01
Comment	
Image Modifications	
