# Supplementary figures and images for: PHF2-mediated H3K9me balance orchestrates heterochromatin stability and neural progenitor proliferation
Source: EMBO Rep. 2024 Jun 18;25(8):18. doi: 10.1038/s44319-024-00178-7 (PMC11315909; doi:10.1038/s44319-024-00178-7)

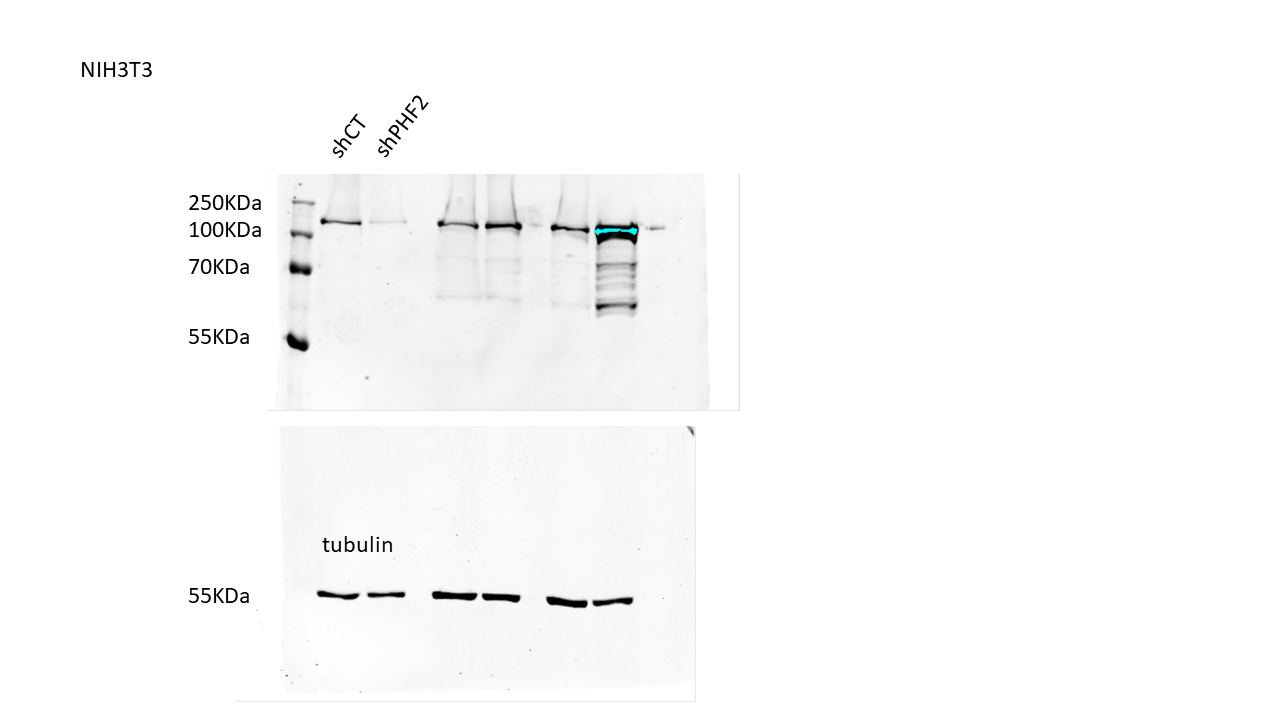

Supplement: Supplementary file 4 — Source data Fig. 2 [file 44319_2024_178_MOESM4_ESM.zip › Figure 2/2B/Blot 2B.TIF]

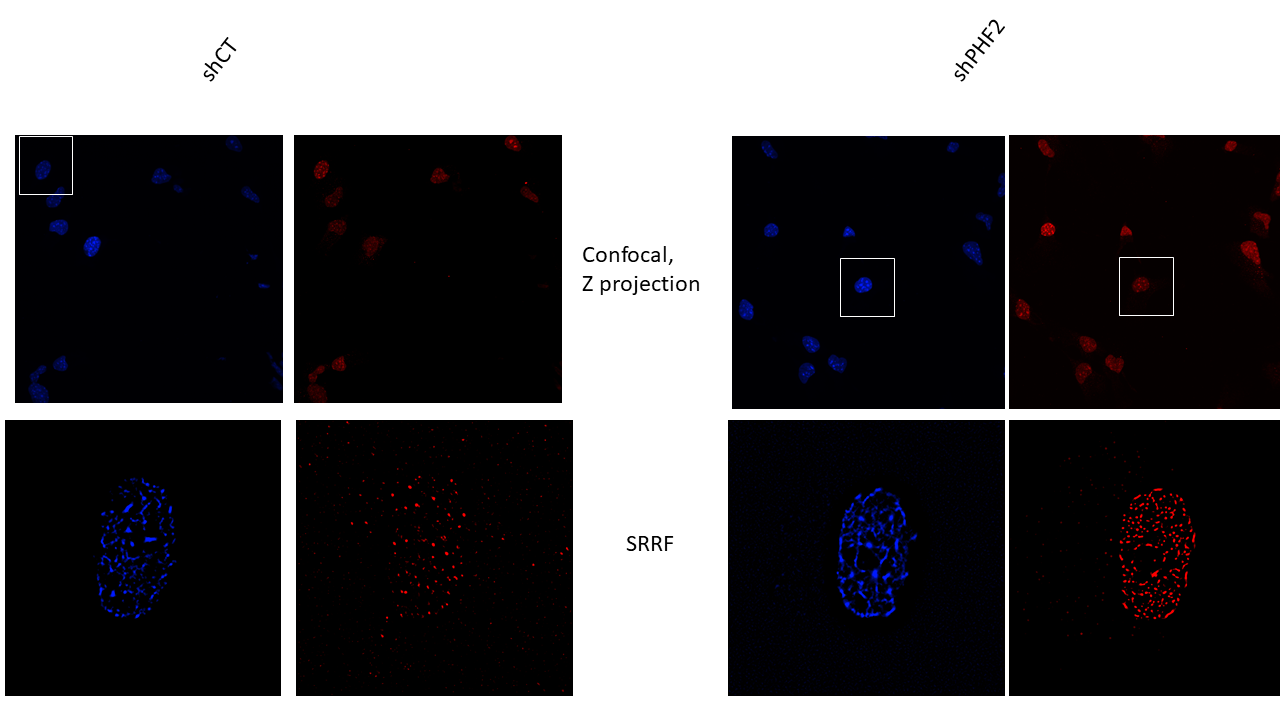

Supplement: Supplementary file 4 — Source data Fig. 2 [file 44319_2024_178_MOESM4_ESM.zip › Figure 2/2C/Image Data NSC 2C.TIF]

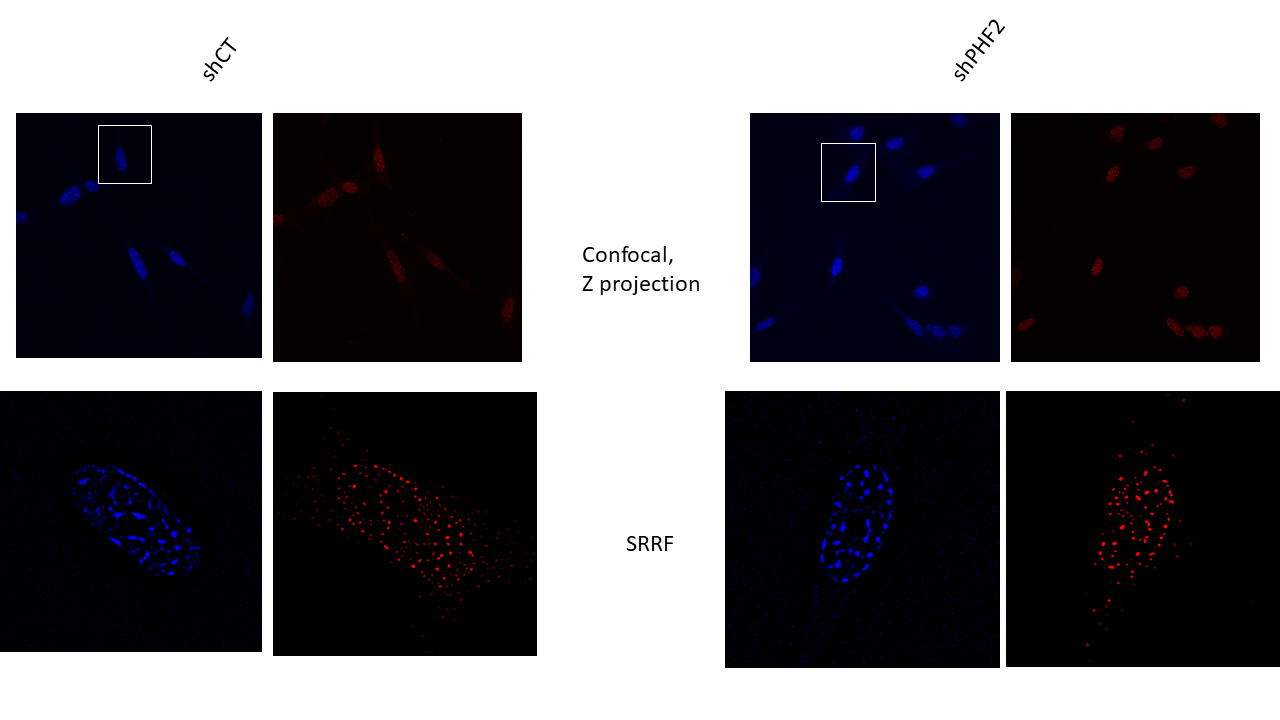

Supplement: Supplementary file 4 — Source data Fig. 2 [file 44319_2024_178_MOESM4_ESM.zip › Figure 2/2D/Image Data NIH3T3 2D.TIF]

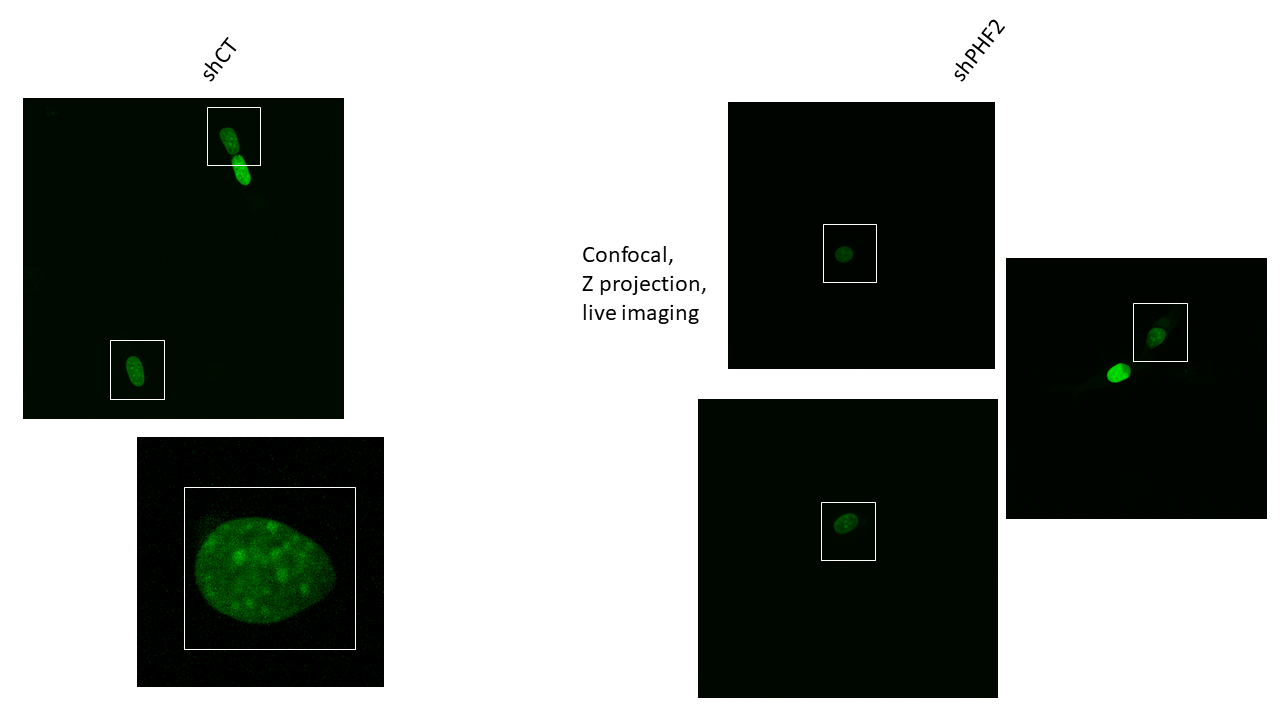

Supplement: Supplementary file 5 — Source data Fig. 3 [file 44319_2024_178_MOESM5_ESM.zip › Figure 3/3G/Image Data 3G.tif]

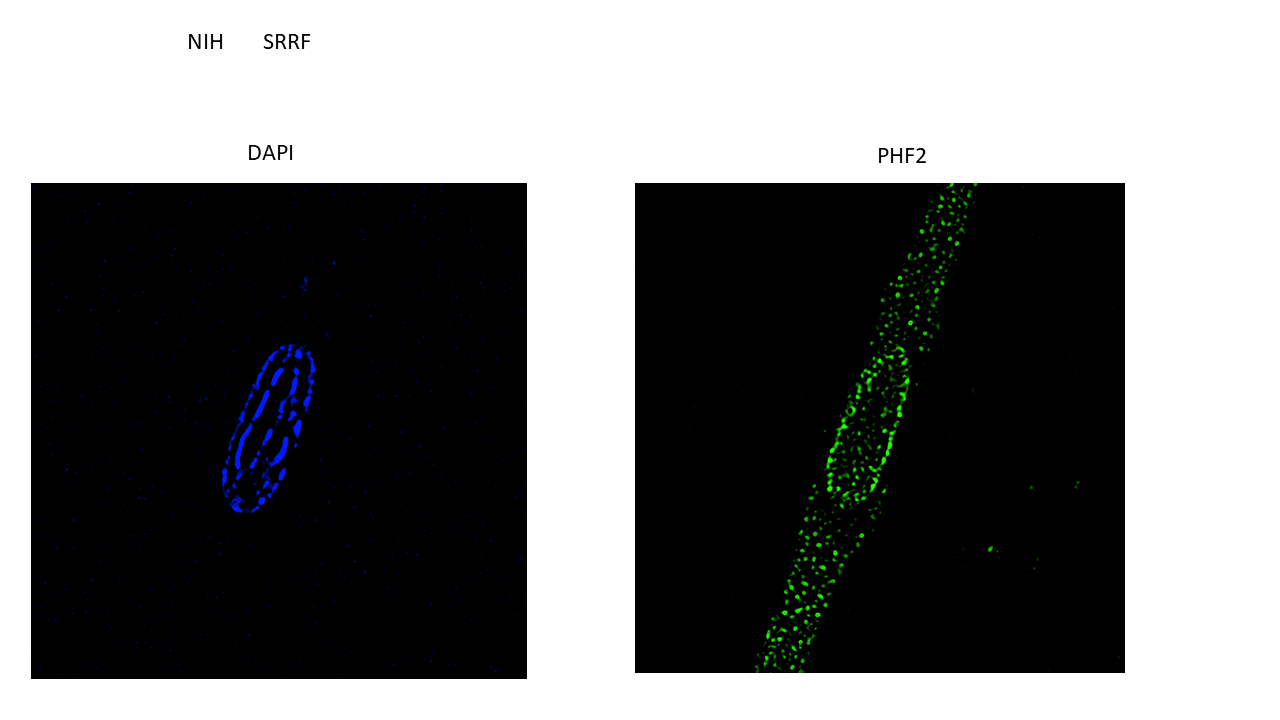

Supplement: Supplementary file 7 — Source data Fig. 5 [file 44319_2024_178_MOESM7_ESM.zip › Figure 5/5D/Image Data 5D.tif]

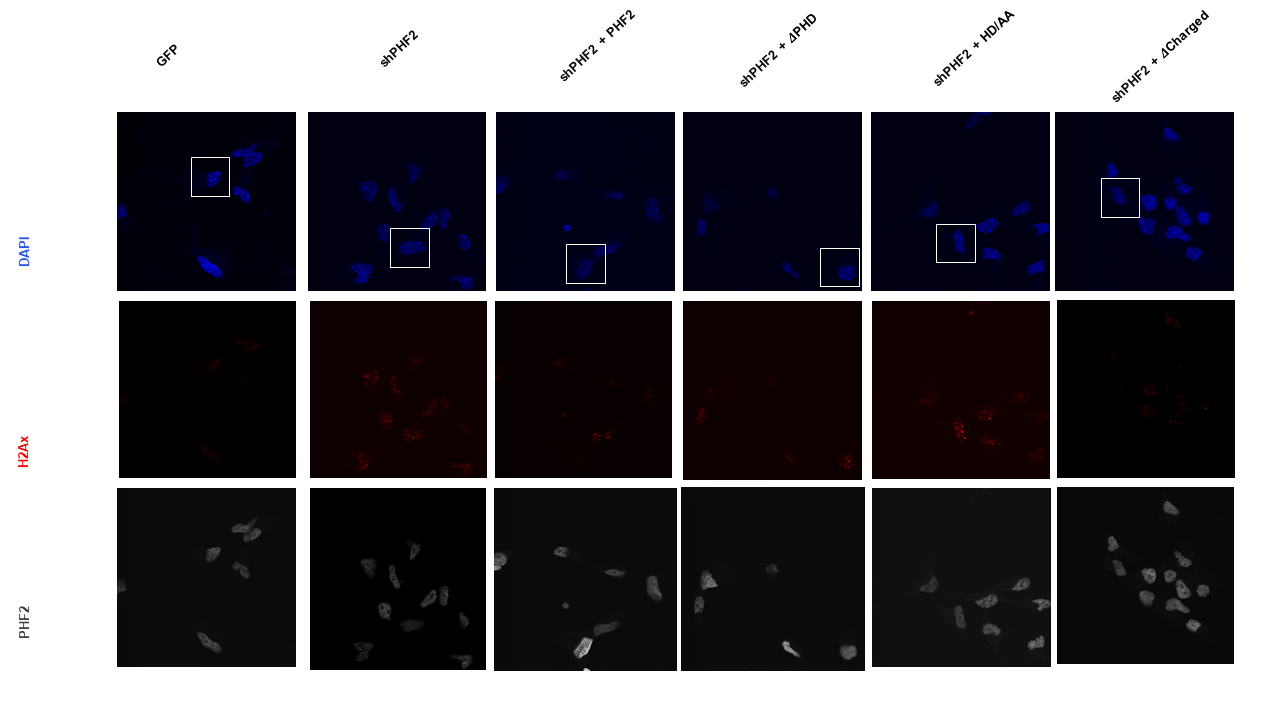

Supplement: Supplementary file 9 — Source data Fig. 7 [file 44319_2024_178_MOESM9_ESM.zip › Figure 7/7C/Image Data 7C.tif]

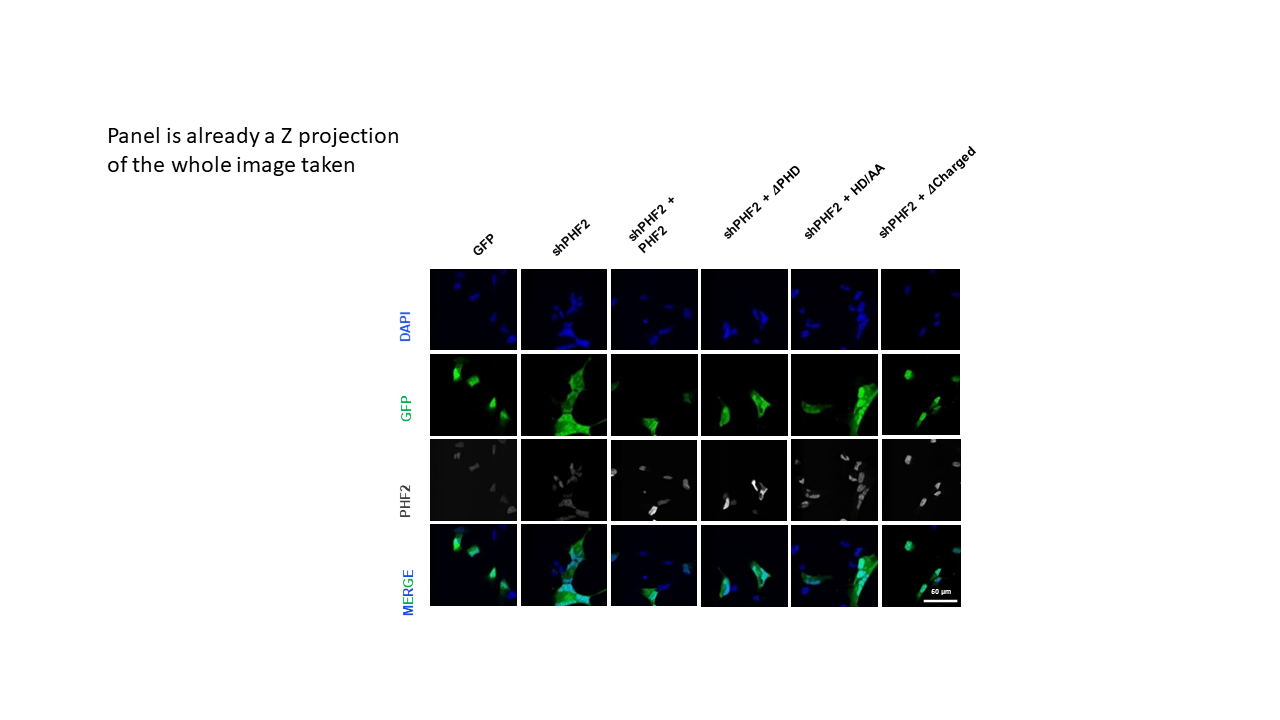

Supplement: Supplementary file 9 — Source data Fig. 7 [file 44319_2024_178_MOESM9_ESM.zip › Figure 7/7D/Image Data 7D.tif]
