# Supplementary material for: PHF2-mediated H3K9me balance orchestrates heterochromatin stability and neural progenitor proliferation
Source: EMBO Rep. 2024 Jun 18;25(8):18. doi: 10.1038/s44319-024-00178-7 (PMC11315909; doi:10.1038/s44319-024-00178-7)
Supplement: Supplementary file 9 — Source data Fig. 7 [file 44319_2024_178_MOESM9_ESM.zip › Figure 7/7A/Blot anti PHF2 mutants.pdf]

Image ID: 0000891_01
Acquire Time: 04-ago-2021 11:56:14

Image Display Values

Channel	Color	Minimum	Maximum	K
700	Gray Scale (Black on White)	2800	46900	0

anti-PHF2

endogenous WT Charged PHD HDAA

250KDa
100KDa
70KDa

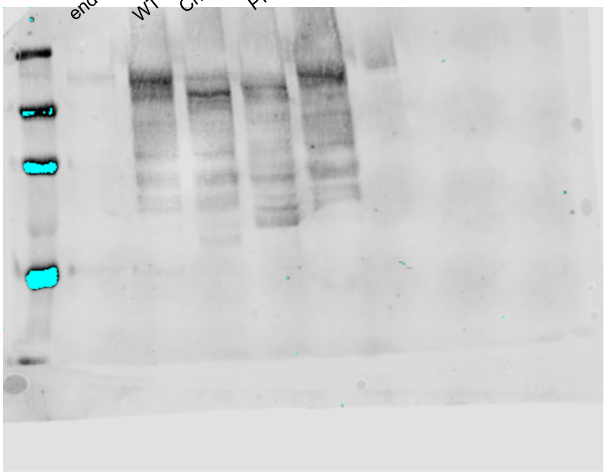

Acquisition Information

Column	Value
Image ID	0000891_01
Acquire Time	04-ago-2021 11:56:14
Channels	700
Resolution	169um
Intensities	8.0
Quality	lowest
Analysis	Manual
Image Name	0000891_01
Comment	
Image Modifications	
