# Supplementary material for: PHF2-mediated H3K9me balance orchestrates heterochromatin stability and neural progenitor proliferation
Source: EMBO Rep. 2024 Jun 18;25(8):18. doi: 10.1038/s44319-024-00178-7 (PMC11315909; doi:10.1038/s44319-024-00178-7)
Supplement: Supplementary file 9 — Source data Fig. 7 [file 44319_2024_178_MOESM9_ESM.zip › Figure 7/7A/Blot anti Tubulin mutants.pdf]

LI-COR®

**Image ID: 0000893_01**
**Acquire Time: 04-ago-2021 13:54:51**



Image Display Values

| Channel | Color | Minimum | Maximum | K |
|---|---|---|---|---|
| 800 | Gray Scale (Black on White) | 92,0 | 3210 | 1 |

55KDa

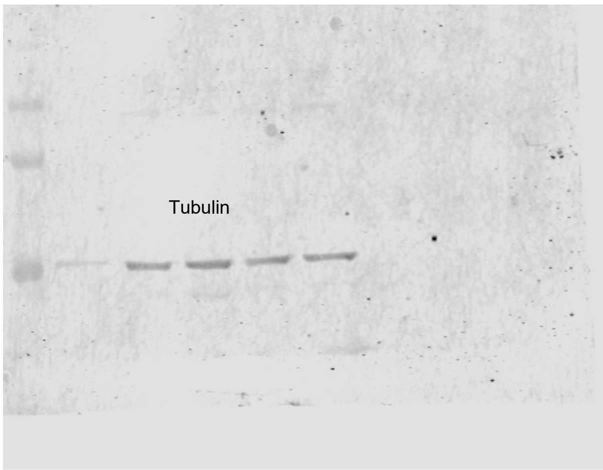


Acquisition Information

| Column | Value |
|---|---|
| Image ID | 0000893_01 |
| Acquire Time | 04-ago-2021 13:54:51 |
| Channels | 800 |
| Resolution | 169um |
| Intensities | 5.0 |
| Quality | lowest |
| Analysis | Manual |
| Image Name | 0000893_01 |
| Comment | |
| Image Modifications | |
