# Supplementary material for: PHF2-mediated H3K9me balance orchestrates heterochromatin stability and neural progenitor proliferation
Source: EMBO Rep. 2024 Jun 18;25(8):18. doi: 10.1038/s44319-024-00178-7 (PMC11315909; doi:10.1038/s44319-024-00178-7)
Supplement: Supplementary file 10 — Expanded View Figures [file 44319_2024_178_MOESM10_ESM.pdf]

Expanded View Figures

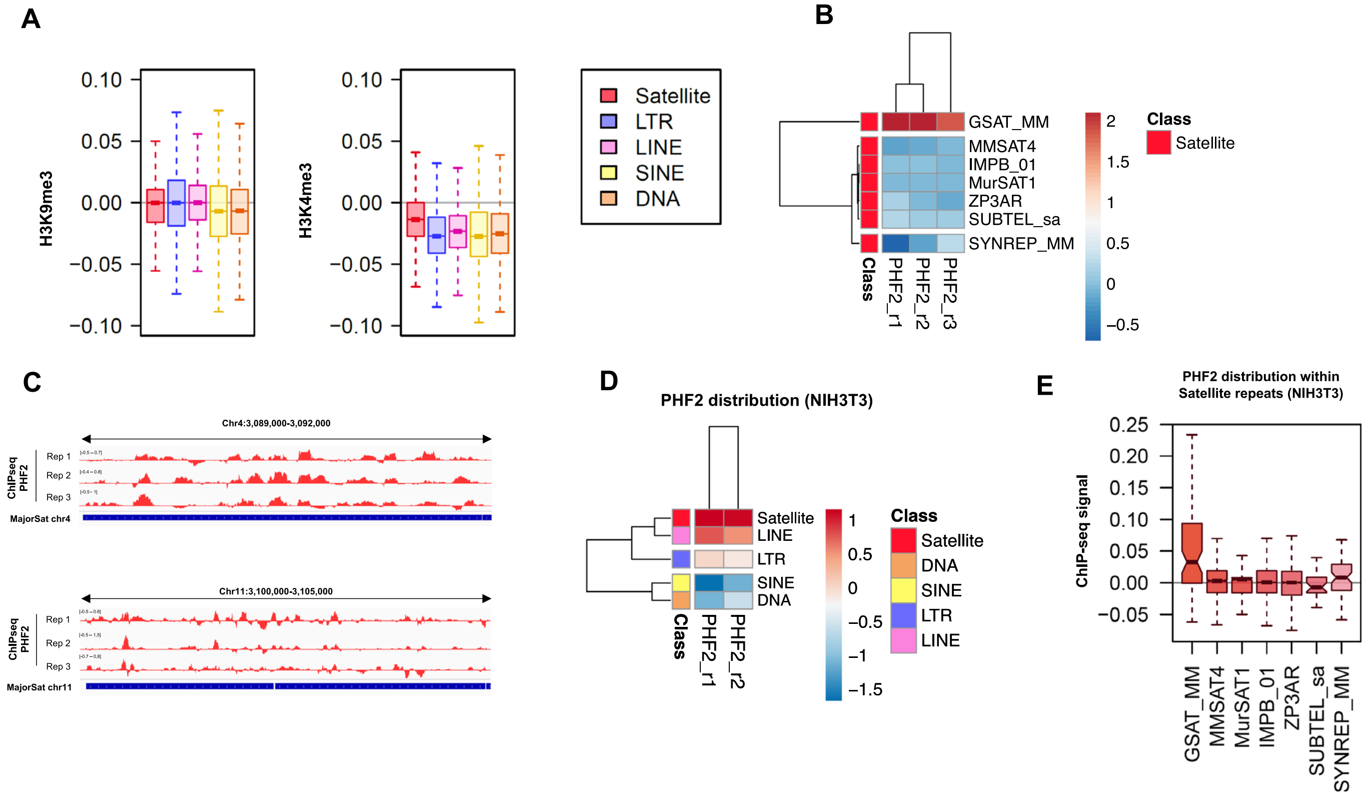

Figure EV1. PHF2 is enriched in satellites repeats.

(**A**) Boxplot showing the H3K9me3 and H3K4me3 input-subtracted ChIP-seq signal within Satellite, LTR, LINE, SINE, and DNA classes of repeats in control NSCs. The boxplots represent the distribution of the different samples' CPM-normalized and input-subtracted ChIP-seq signal (y-axis) in the corresponding group of repeats (x-axis). Box plots: centerlines show the medians; box limits indicate the 25th and 75th percentiles; whiskers extend to the minimum and maximum. Data used for this analysis correspond to the averaged values obtained from two independent ChIP-seq replicates. Exact sample sizes for each group: Satellite = 36234; LTR = 970039; LINE = 987285; SINE = 1527608; DNA = 162787. (**B**) Heatmap and clustering of the mean input-subtracted ChIP-seq signal of three independent PHF2 replicates within seven groups of repeats belonging to the Satellite class. Box plots: centerlines show the medians; box limits indicate the 25th and 75th percentiles; whiskers extend to the minimum and maximum. Exact sample sizes for each group: GSAT_MM = 77; MMSAT4 = 1597; MurSAT1 = 5398; IMPB_01 = 26485; ZP3AR = 2570; SUBTEL_sa = 31; SYNREP_MM = 69. (**C**) IGV genome browser screenshots illustrating the continuous input-subtracted quantification of three replicates of PHF2 ChIP-seq samples within the major satellite of chromosmes 4 and 11. (**D**) Heatmap and clustering of the mean input-subtracted ChIP-seq signal of two independent PHF2 replicates in NIH3T3 cells in Satellite, DNA, SINE, LTR, and LINE classes of repeats. (**E**) Boxplot showing the PHF2 input-subtracted ChIP-seq signal in NIH3T3 cells within seven groups of repeats belonging to Satellite class in shCT condition. The boxplots represent the distribution of the different samples' CPM-normalized and input-subtracted ChIP-seq signal (y-axis) in the corresponding group of repeats. Box plots: centerlines show the medians; box limits indicate the 25th and 75th percentiles; whiskers extend to the minimum and maximum. Data used for this analysis correspond to the averaged values obtained from two independent PHF2 ChIP-seq replicates in NIH3T3 cells. Exact sample sizes for each group: GSAT_MM = 77; MMSAT4 = 1597; MurSAT1 = 5398; IMPB_01 = 26485; ZP3AR = 2570; SUBTEL_sa = 31; SYNREP_MM = 69.

© The Author(s)

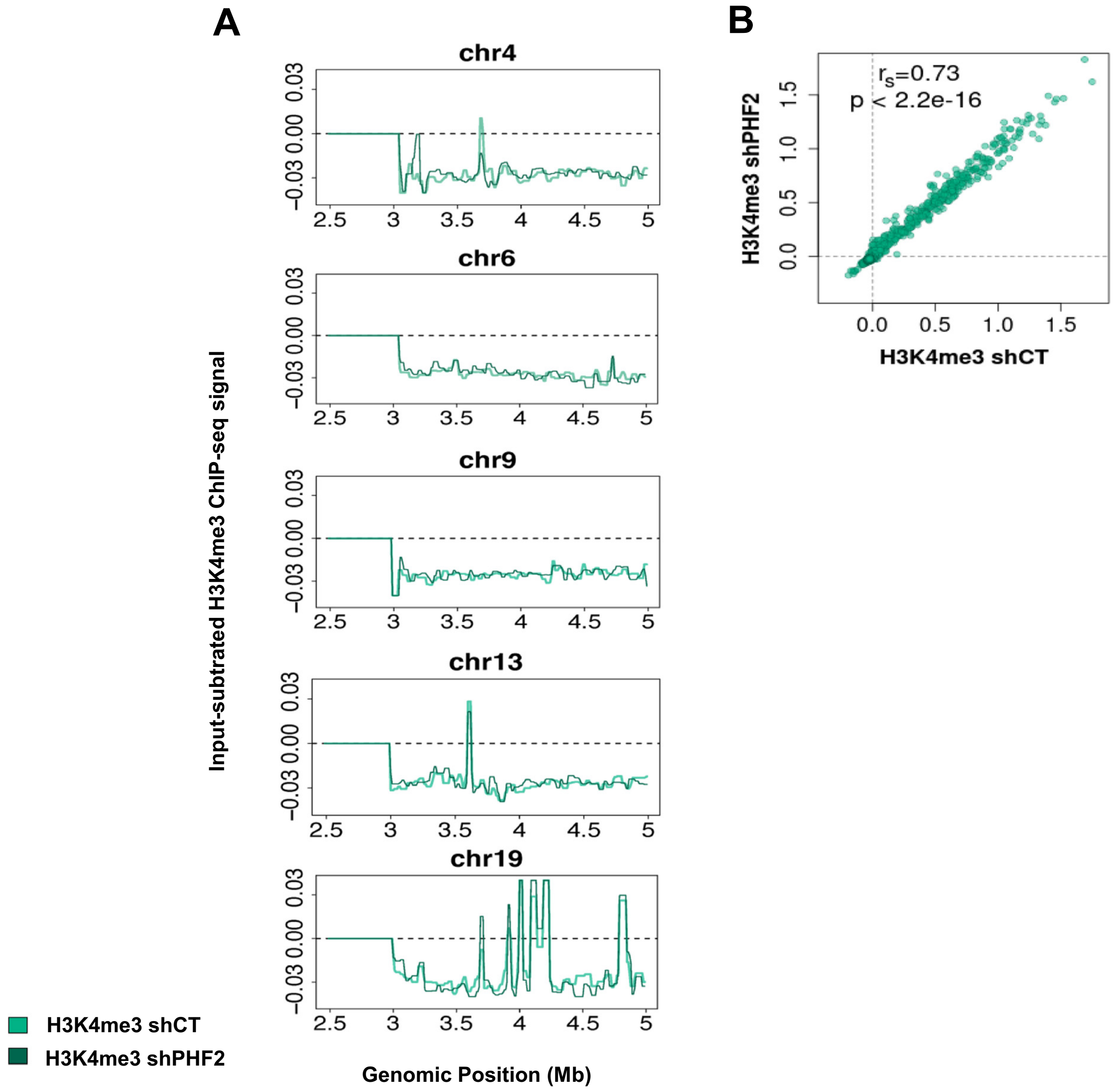

Figure EV2. PHF2 balances H3K9me3 at Pc boundaries.

(**A**) Line plot showing the H3K4me3 profile across the first 5 Mb (2.5–5 Mb) from the centromeric end of chromosomes 4, 6, 9, 13, and 19 in control and PHF2-depleted NSCs. Data used for this analysis correspond to the averaged input-subtracted signal obtained from two independent H3K4me3 ChIP-seq replicates. (**B**) Spearman's correlation between H3K4me3 input-substracted ChIP-seq signal in control and PHF2-depleted NSCs. Data used for this analysis correspond to the averaged input-subtracted values within 10 Kb bins along the whole genome obtained from two independent H3K4me3 ChIP-seq replicates.

 © The Author(s)

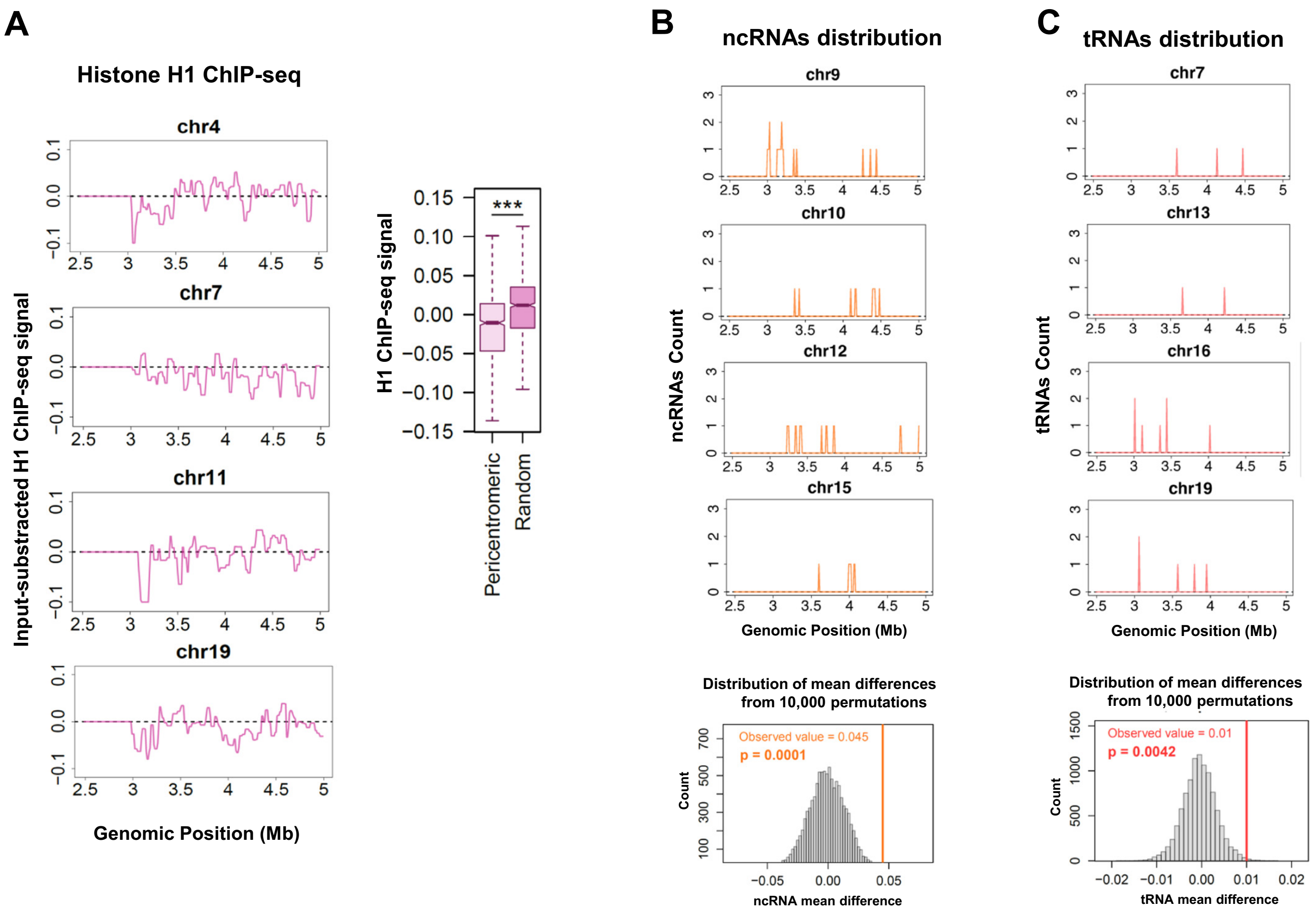

Figure EV3. PcH boundaries characterization.

(**A**) Line plot showing the histone H1 (GSM1199586) profile across the first 5 Mb (2.5–5 Mb) from the centromeric end of chromosomes 4, 7, 11, and 19 in mESCs. Data used for this analysis correspond to the averaged input-subtracted ChIP-seq signal (left panel). Boxplot displaying the histone H1 input-subtracted ChIP-seq signal within 1000 pericentromeric bins (3–3.5 Mb) from all chromosomes compared to 1000 random bins. The boxplots represent the distribution of the sample's CPM-normalized and input-subtracted ChIP-seq signal (y-axis) in the corresponding group of bins (x-axis). Box plots: centerlines show the medians; box limits indicate the 25th and 75th percentiles; whiskers extend to the minimum and maximum. ***$p < 0.001$ (Mann- Whitney U test). Exact sample sizes for each group: Pericentromeric = 1000; Random = 1000 (right panel). (**B**) Line plot showing the count distribution of ncRNAs (Ensembl GRCm38.86 annotation) across the first 5 Mb (2.5–5 Mb) from the centromeric end of chromosomes 9, 10, 12, and 15 (upper panel). Histogram of the mean differences from the permutation test between ncRNAs count distribution in pericentromeric and random bins. A total of $N = 10{,}000$ permutations were performed. The orange vertical bar corresponds to the observed difference in medians between ncRNAs count distribution in pericentromeric and random bins (lower panel). (**C**) Line plot showing the count distribution of tRNAs (Genomic tRNA Database, GtRNAdb, annotation) across the first 5 Mb (2.5–5 Mb) from the centromeric end of chromosomes 7, 13, 16, and 19 (upper panel). Histogram of the mean differences from the permutation test between tRNAs count distribution in pericentromeric and random bins. A total of $N = 10{,}000$ permutations were performed. The pink vertical bar corresponds to the observed difference in medians between tRNAs count distribution in pericentromeric and random bins (lower panel).

© The Author(s)

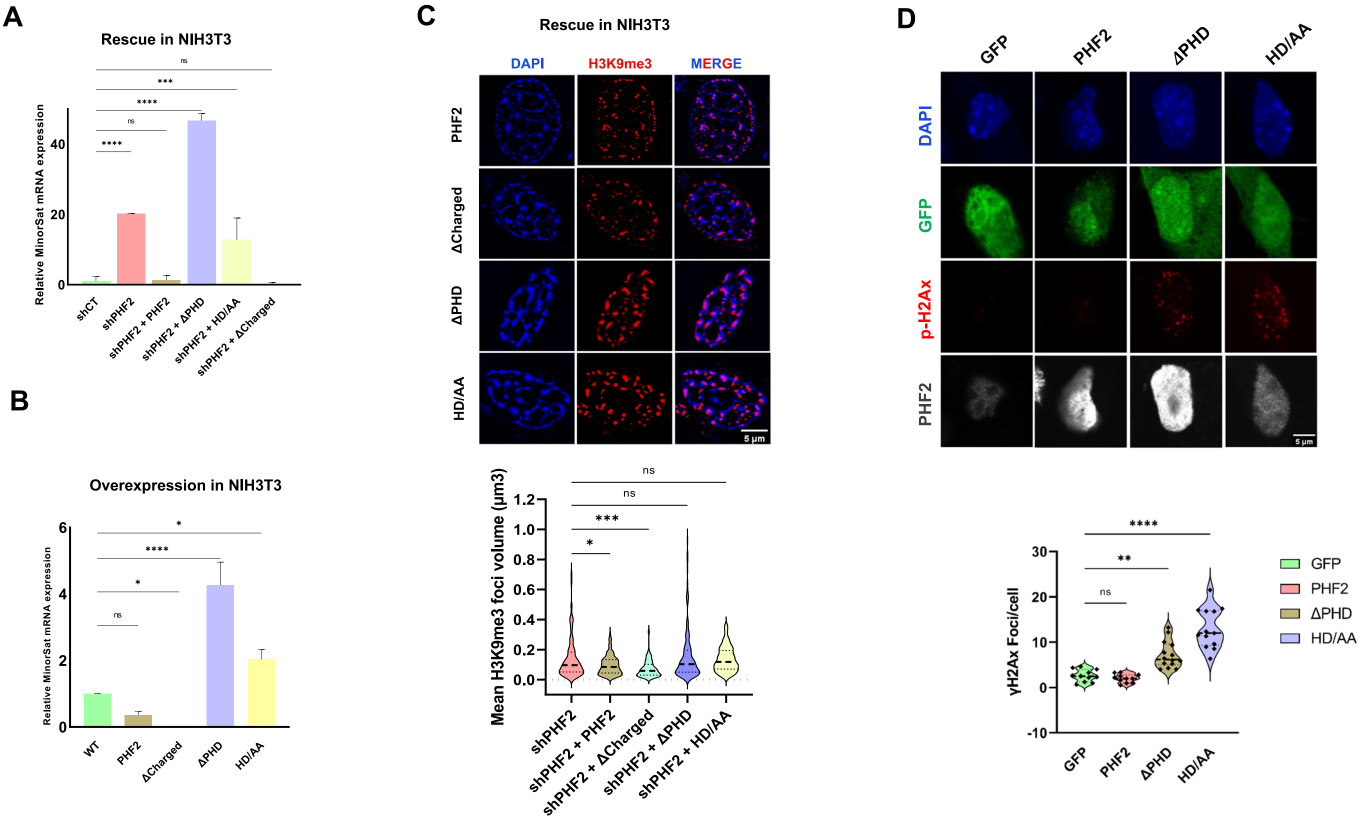

Figure EV4. The maintenance of PcH stability relies on the PHD and JmjC domains within PHF2.

(**A**) PHF2-depleted NIH3T3 were transfected with PHF2 WT, ΔCharged, ΔPHD, or HD/AA mutants. Total RNA was prepared and the levels of RNA levels of major satellite, minor satellite, were determined by qPCR. Mean was calculated from data of 3 biologically independent experiments and normalized to *Gapdh* reference gene levels and figure shows values relative to shCT cells. Error bars indicate SD. ***$p < 0.001$, ****$p < 0.0001$ (Student's t-test). (**B**) NIH3T3 cells were transfected with PHF2 WT, ΔCharged, ΔPHD, or HD/AA mutants. Total RNA was prepared and the levels of RNA levels of major minor satellite, were determined by qPCR. Mean was calculated from data of 3 biologically independent experiments and normalized to *Gapdh* reference gene levels and figure shows values relative to shCT cells. Error bars indicate SD. *$p < 0.05$, ***$p < 0.001$ (Student's t-test). (**C**) PHF2-depleted NIH3T3 cells were transfected with PHF2 WT, ΔCharged, ΔPHD, or HD/AA mutants. Super resolution (SRRF) immunostaining is depicted using anti H3K9me3 antibody and DAPI. The volume of the H3K9me3 foci were determined on confocal images using ImageJ software (see Methods). Data shown are representative of three biologically independent experiments. Scale bar indicates 5 µm. Violin plots represent the foci volume quantification of H3K9me3 foci of 100 cells ($n = 100$). *$p < 0.05$; ***$p < 0.001$ (Student's t-test). (**D**) NSCs were nucleofected either with GFP alone or with PHF2 WT, ΔPHD, or HD/AA mutants. Cells were fixed and stained with PHF2 and γH2Ax antibodies and DAPI. Green cells were analyzed. More than 30 cells were quantified. Data shown are representative of 3 biologically independent experiments. Scale bar indicates 5 µm. Violin plots represent the number of γH2Ax foci/cell. **$p < 0.01$, ****$p < 0.0001$ (Student's t-test).

 © The Author(s)
